# Supplementary figures and images for: The high diversity of gametogenic pathways in amphispermic water frog hybrids from Eastern Ukraine
Source: PeerJ. 2022 Aug 23;10:e13957. doi: 10.7717/peerj.13957 (PMC9415524; doi:10.7717/peerj.13957)

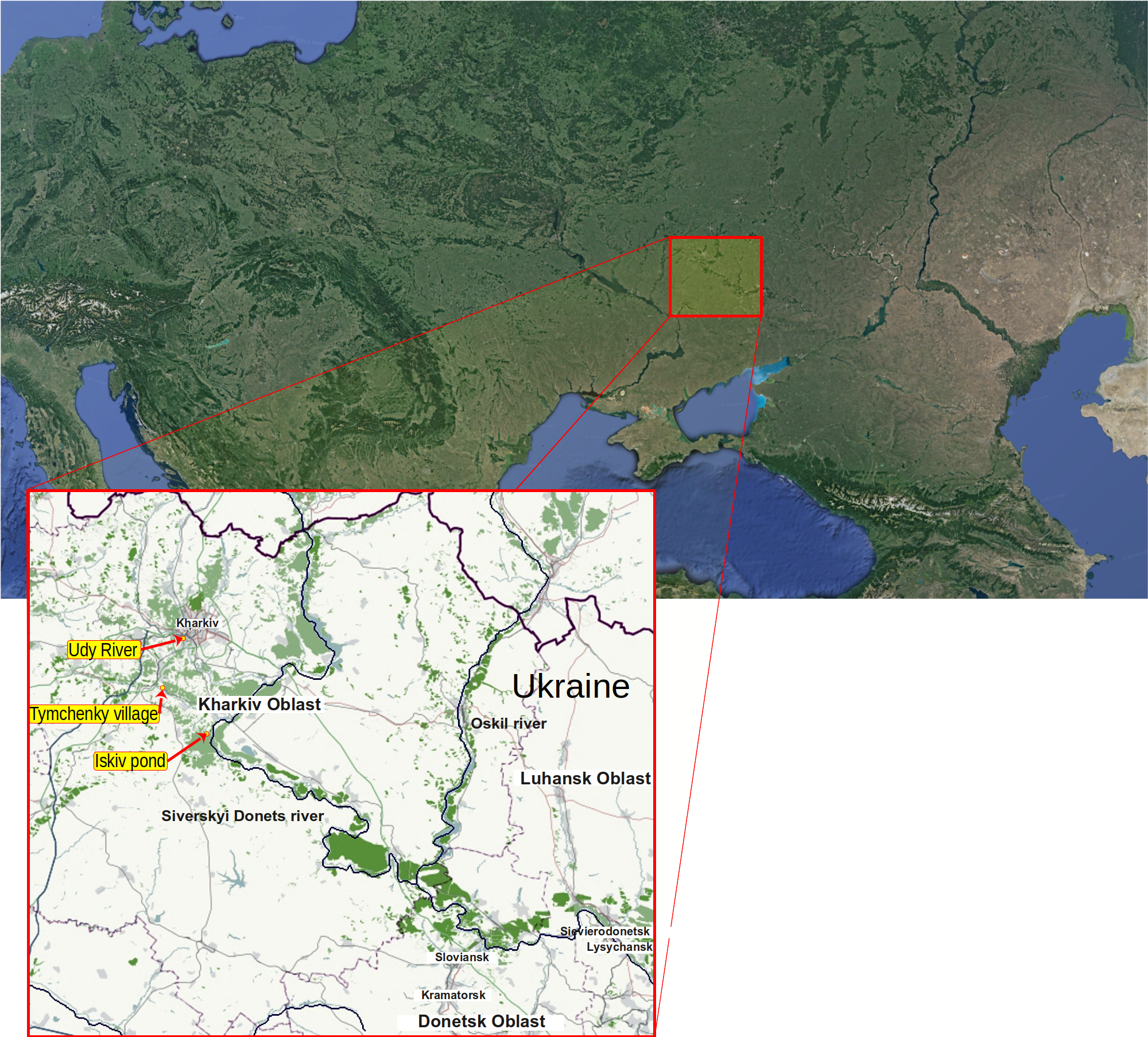

Supplement: Figure S1 [file peerj-10-13957-s001.png]

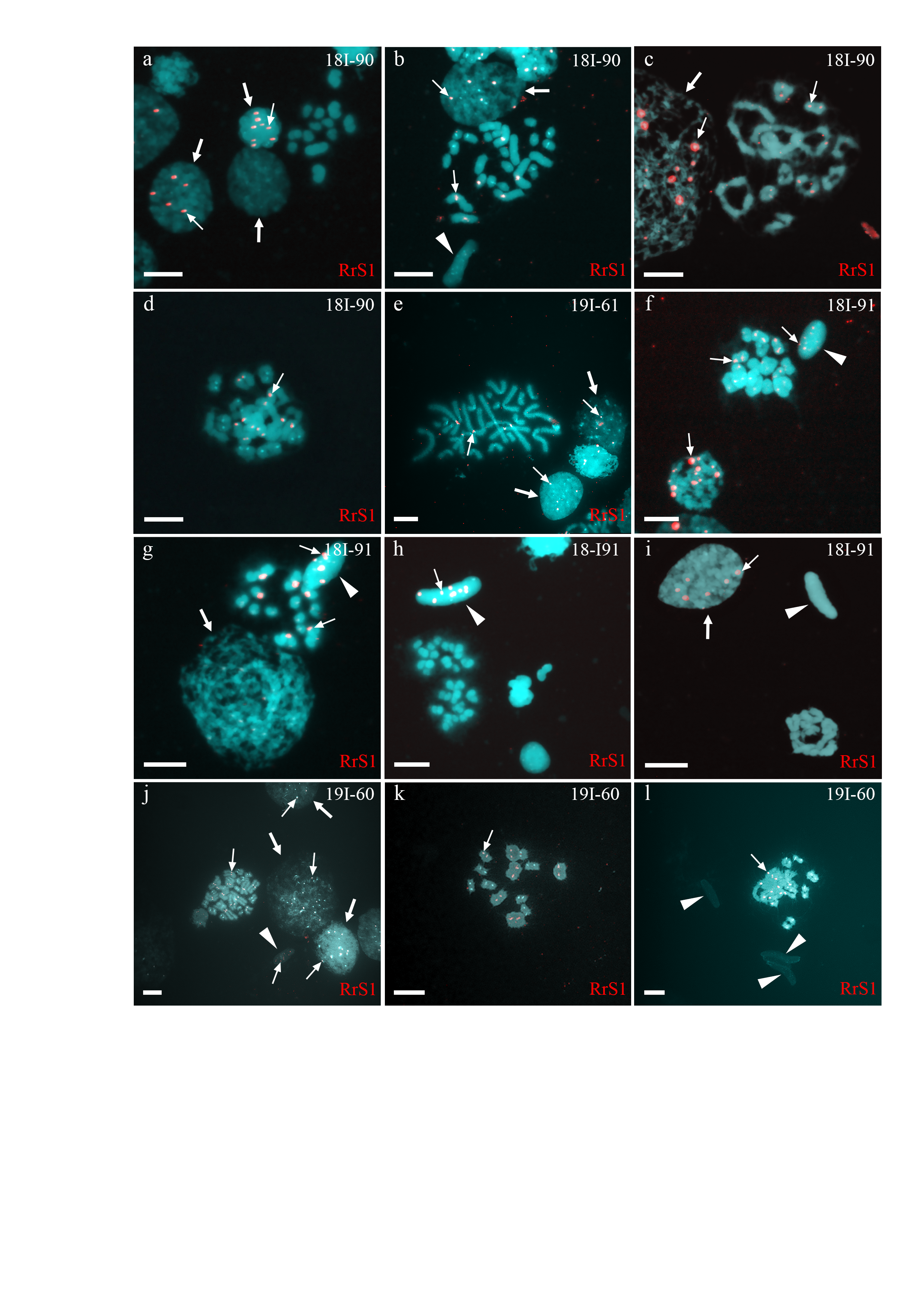

Supplement: Figure S2 — FISH with RrS1 probe helped distinguish pericentromeric regions only of P. ridibundus chromosomes (indicated by thin arrows). Interphase cell nuclei (indicated by thick arrows) with haploid P. ridibundus (A-C, E, F, I, J) or P. lessonae (A, G, J) chromosomal sets. Mitotic metaphases with 26 chromosomes each of P. ridibundus and P. lessonae (B, E) and with only P. ridibundus chromosomes (J). Meiotic metaphase II with 13 univalents of P. lessonae (A, H) and 13 univalents of P. ridibundus (G). Meiotic metaphase I with 13 bivalents of P. ridibundus (C, D, F, K, L). Spermatids (indicated by arrowheads) with haploid sets of P. ridibundus chromosomes (F-H) and P. lessonae (B, I, L). Scale bar = 10µm [file peerj-10-13957-s002.png]

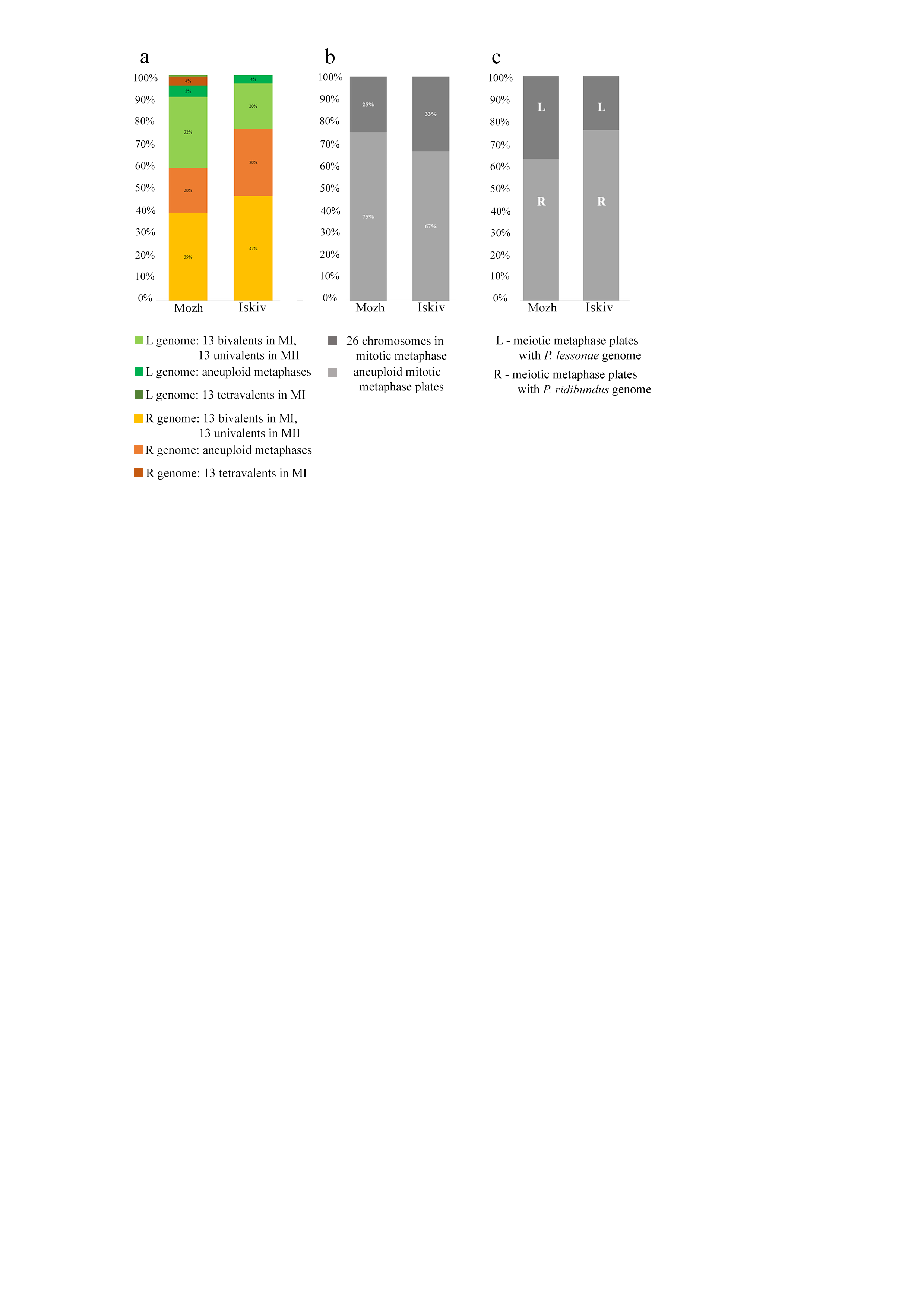

Supplement: Figure S3 — The proportion of meiotic plates with different genomes from hybrid frogs collected from the R-E system of the Mozh river (left column) and Iskiv pond (right column). R –genome of P. ridibundus, L –genome of P. lessonae; aneuploidy –number of chromosomes more or less 13 bivalents or univalents. [file peerj-10-13957-s003.png]
